# 6. FACT OR ARTIFACT? BENEFITS AND LIMITATIONS OF ADVANCED NEUROIMAGING METHODS FOR PSYCHOSIS

**DOI:** 10.1093/schbul/sby014.017

**Published:** 2018-04-01

**Authors:** Ofer Pasternak

**Affiliations:** Harvard Medical School

## Abstract

**Overall Abstract:** Neuroimaging tools introduced the ability to non-invasively search for pathological signatures in the brains of subjects suffering from psychosis. In fact, with almost any neuroimaging modality there are studies that report the identification of abnormalities in the brains of schizophrenia subjects. The abundance of findings made it much clearer that brain abnormalities are common and expected in mental disorders. However, the quantity of findings, and especially their inconsistence across studies also raise questions as to the source of these abnormalities. Are they signifying a complicated range of pathologies and interactions? Or do they reflect auxiliary changes that are not directly related to the root, or the etiology of the disorder?

In addition, these inconsistencies raise technical questions such as are our tools sufficiently sensitive to reliably identify brain abnormalities in psychosis? And, to what extent are our tools sensitive to misinterpretation and to artifacts, which may explain some of the group differences found when comparing psychotic populations with controls? Emerging imaging modalities attempt to address these concerns by improving the specificity, i.e., the ability to relate identified abnormalities with underlying pathologies. At the same time analysis must carefully pay attention to common physiological sources of artifacts, such as subject motion, blood flow, brain metabolism, partial volume, etc.

This symposium will bring together five leading neuroimagers from 4 continents, who will present paradigm-shifting state of the art in their field, while providing critical cautionary remarks regarding the shortcomings of these methods, as well as recommendations for proper use in the context of psychosis studies. Speakers are:

1) Prof. Jennifer Caughlin, M.D. Department of Psychiatry and Behavioral Sciences, Johns Hopkins University. She will present state of the art in TSPO-PET acquisition and analysis, and its potential for the evaluation of neuroinflammation in schizophrenia.

2) Prof. Ofer Pasternak, Ph.D. Departments of Psychiatry and Radiology, Harvard Medical School. He will present recent advances in diffusion MRI, and how microstructural imaging may inform the study of psychosis.

3) Prof. Christoph Mulert, M.D. Department of Psychiatry and Psychotherapy. University medical center Hamburg, Germany. He will present new approaches for EEG acquisitions combined with fMRI that may shed light on neuronal activity.

4) Prof. Helen Juan Zhou, Ph.D. Neuroscience & Behavioural Disorders Programme, Duke-Nus Medical School, Singapore. She will present the emerging field of connectomics and its potential application to study functional and structural brain networks in schizophrenia.

The discussant will be Prof. Christos Pantelis, M.D. Department of Psychiatry, University of Melbourne, Australia. Dr. Pantelis will complement the panel by bringing in a more clinical point of view, informed of the needs in psychiatric neuroimaging. The panel will also benefit from his vast experience across all imaging modalities.

This symposium is designed to provide information and considerations that can be essential for psychosis researchers before considering the application of advanced imaging tools. We will describe the main and unique findings that were provided by each modality, towards a discussion of how far are imaging studies from leading to a breakthrough in the understanding of the pathophysiology of psychosis or its treatment.

